# Estimating a treatment effect on recidivism for correctional multiple component treatment for people in prison with an alcohol use disorder in England

**DOI:** 10.1186/s13011-020-00310-5

**Published:** 2020-10-15

**Authors:** Arun Sondhi, Alessandro Leidi, David Best

**Affiliations:** 1 Therapeutic Solutions (Addictions) Ltd, London, UK; 2 Statistical Services Centre, Reading, UK; 3 University of Derby, Derby, UK

**Keywords:** Alcohol use disorder, Prisons, Multiple component interventions, Multiple treatments, Reoffending

## Abstract

**Background:**

There is an emerging literature on the impact of correctional substance abuse treatment (SAT) on reoffending for people in prison with substance misuse issues. This study estimates a pathway effect for people in prison receiving multiple component treatments for an alcohol use disorder (AUD) to reduce reoffending by applying treatment effect estimation techniques for observational studies. Treatment groups comprised pharmacological treatments, psychosocial interventions (PSIs) and interventions that incorporate Risk Need Responsivity (RNR) programming. RNR compliant treatment matches treatment dose to the risk of reoffending, targets criminogenic need and is tailored to a person’s learning style.

**Methods:**

Multiple treatment effect estimators are provided for people in prison diagnosed with an AUD in England when compared to a derived control group for: Pharmacological treatment only; RNR compliant treatment and PSIs.

**Results:**

The outcomes for RNR compliant treatment suggest a lower recidivism rate compared to the control group. Pharmacological only treatment results in a statistically significant higher level of reoffending relative to the control group.

**Conclusions:**

The creation of a universal system of ‘equivalence of care’ framed within a public health context in English correctional SAT may have had an unintended consequence of diluting approaches that reduce recidivism. There is an opportunity to develop an integrated, cross-disciplinary model for correctional SAT that unites public health and RNR compliant approaches.

## Background

Studies have highlighted the association between violence and alcohol with high prevalence estimates of alcohol use disorders (AUD) noted in correctional settings [19]. International estimates for AUDs among recently incarcerated people in prison are around one-quarter (24%) compared to 30% illicit drug use for males and around half (51%) for females [18]. In England, just under half (48%) of people in prison accessing substance misuse treatment services will have a diagnosed AUD, of which around one in ten (11%) were misusing alcohol only and not illicit drugs [44]. Evaluations of correctional substance abuse treatment (SAT) have suggested positive effects on reoffending through reductions in reoffending rates [8, 21, 39, 40, 42, 52, 56]. These positive findings have also been demonstrated for AUD clients in community treatment [55] although poorer outcomes relative to illicit drug misusers have been noted [22]. Although there is a sizeable literature on SAT in correctional settings whereby treatment for AUDs has been integrated within interventions addressing illicit drug misuse, there is relatively little known on the effectiveness of correctional treatment for people in prison who present with alcohol issues only. Commentators have argued that there is a need to focus on outcomes for drinkers which has been considered an overlooked area in treatment effectiveness research [17, 22].

In England, correctional SAT encompasses a variety of treatment styles and philosophies offered to differing offender segments. The UK Government’s Drug Strategy [25] emphasised offenders’ access to SAT at all stages of the criminal justice system with an explicit aim to reduce reoffending [38]. The National Health Service (NHS) supports English community and correctional SAT. Implicit in this framework is the concept of ‘equivalence of care’ across correctional and community SAT based on the principle of universality, where any person in prison is entitled to access the same treatment in correctional settings as in the community. Despite this aim, inconsistencies in achieving equivalence of care have been noted due to the increased size and complexity of the prison population, alongside the effects of austerity on resourcing [28].

Although correctional SAT is conceptualised as a single treatment system, there are a number of multicomponent interventions including pharmacological treatments that manage symptoms of withdrawal (acamprosate, disulfiram or naltrexone). Access to prison-based SAT can occur across jurisdictions through a medical assessment of alcohol-related needs including severity of consumption, and a criminal behavioural assessment by offender managers who refer onwards to SAT if problematic use has been identified. The approach to delivering SAT is underpinned by national guidelines on clinical management [14, 37].

Historically in English correctional settings, interventions were developed using structured (or ‘manualised’) group programs incorporating Risk Need Responsivity (RNR) components [2]. Andrews and Bonta [1] have highlighted the three principles underpinning effective treatment programs aimed at offender rehabilitation. The first principle focuses on an offender’s likelihood of offending risk, such that higher-risk offenders are required to receive more intensive (higher ‘dose’) interventions. The second facet is to address the criminogenic and non-criminogenic needs of an offender. These are subdivided into static (age, gender, ethnicity, offending history) and dynamic factors (motivation, attitude to authority etc.). The final component is ‘responsivity’ including ‘general’ responsivity that posits cognitive-behavioural interventions are more efficacious than nonbehavioral approaches [2]. Interventions that incorporate these components will be defined as ‘RNR compliant’ treatment. The combination of pharmacological treatment and psychosocial interventions (PSIs) are provided within each prison across England with an emphasis on establishing a therapeutic alliance between therapist and client through evidence-based practices including cognitive behavioural therapies that focus on alcohol consumption that trigger criminal behaviours [14]. Behavioural programs for offenders have been largely decommissioned, replaced by a range of PSIs including one-to-one cognitive therapies and structured group work with peer or mutual aid support offered where possible [54]. PSIs are less likely to be focused on the offence (e.g. violent behaviour or drink-driving) but rather address remission by tackling alcohol-related cognitions and its relationship to criminal behaviour. These interventions will not be primarily based on RNR principles and therefore will be classified as ‘PSIs’.

The aim of the study is to estimate a treatment effect on recidivism for multiple component treatment for people in prison with an AUD in England. The components of SAT to reduce recidivism will be compared to each other and to a control group. This excludes those who use illicit drugs and focuses on AUD needs only. The analysis will estimate a treatment effect for three interventions: pharmacological treatment, RNR compliant and other PSIs. The paper will contribute to the literature of estimation of treatment effectiveness in the context of observational studies, by utilising regression-based methods to estimate the effect of multiple treatment. Few studies have been able to determine a treatment effect where there are competing interventions, thereby limiting any explanation underpinning the efficacy of a single treatment system or program [51].

## Methods

Data for this study were derived from an administrative dataset of 59,150 adult prison leavers (aged 18 or more years) in England released into the community during 2013–14. Reconviction data was collected on each prisoner during 2014–15 and separately incorporated into the dataset. Data on prison leavers were linked to the National Drug Treatment Monitoring System (NDTMS) a public health surveillance system used in the community and in prison settings. Where multiple records of a treatment episode existed, the one nearest to the date of release from prison was chosen. A total of 2647 people in prison (10%) reported to NDTMS as having received treatment for an AUD and released during 2013–14 were matched with the prison leavers dataset using a common unique identifier (‘NOMSID’). People in prison identified as having an AUD but not known to NDTMS and therefore not exposed to prison-based SAT were considered part of the control group (*n* = 24,007).

The possibility of whether any people in prison reported within the control group had received AUD treatment (for example, not recorded due to data entry error) was examined through combining the unique ID match with ‘fuzzy logic’ linkages based on personal identifiers held across both datasets (initials, date of birth and gender). An initial run generated around 8000 possible matches but adjusting the logic to ‘likely’ characteristics (e.g. gender, ethnicity, home location) matched to the same prison and time period generated 166 possible matches (1%) where a person may have received AUD treatment. Sensitivity tests using the single and enhanced matches generated very similar outcomes and therefore the unique identifier match was used as the final analytical method.

Details on the treatment received are captured on NDTMS and included within a ‘modality’ variable [45]. Here interventions are categorised using broad headings such as ‘pharmacological interventions’ for pharmacological treatment, and five interventions that are grouped under a wider PSI heading. These include ‘counseling’; ‘cognitive behavioural therapy’; ‘motivational interviewing’; ‘relapse prevention’ and ‘family work’. An initial exploration of these groupings found that people in prison received a number of PSIs in combination over one episode of treatment. As there were multiple and disparate therapies, these were grouped into a single PSI component to boost sample size. 241 (9%) people in prison were coded as receiving RNR compliant treatment in that they were categorised as high-risk offenders receiving the most intensive treatment dose (dosage was calculated as the length of treatment for that treatment episode coded into tertiary bands of low-medium-high levels) whilst also receiving one of the five evidence-based PSIs highlighted above. Seven hundred thirty three people in prison (28%) received pharmacological treatment only with no PSI input and 1673 (63%) people in prison received PSIs.

### Participants

A summary of the crude (unadjusted) characteristics by socio-demographic and offending details of the control and treatment groups is presented in Table 1. The offence data show the mean number of offences for people in prison by control or treatment group, alongside the mean length of stay (spell length) in prison for each group.

**Table 1:** Unweighted characteristics of the control and treatment groups

Prognostic	Control (Crude) [n = 24,007]		Treated (Crude) [***n*** = 2647]	
	Number	Percentage	Number	Percentage
Male	22,507	93.8%	2402	90.7%
	Mean	SD	Mean	SD
Age, mean (SD)	31.86	(9.56)	32.5	(10.7)
	Mean		Mean	
Age at first offence (SD)	16.2	(5.9)	19.3	(8.8)
Previous Court Order	5.7		5.0	
Breach of Order	4.6		4.1	
Previous Offences	44.3		43.5	
Previous Prison Events	6.6		6.6	
Previous Convictions	19.5		20.9	
Criminal Damage	2.8		3.1	
Burglary	1.5		1.0	
Drink Driving	0.6		0.8	
Drugs import/export	0.2		0.1	
Drugs Possession	1.8		1.1	
Fraud	0.9		0.9	
Handling	0.9		0.5	
Other Burglary	1.7		1.4	
Public Order	3.1		5.7	
Robbery	0.4		0.3	
Sexual Offences	0.1		0.2	
Theft	8.8		8.0	
Theft of Vehicle	0.8		0.4	
Violence against Person	5.4		6.7	
Severity of Offence (low)	40.8		41.0	
Spell length (weeks)	51.6		39.2	

### Outcome measure

A binary definition of reoffending (yes/no) for people in prison released during 2013–14 was calculated from the Police National Computer that included any individual with at least one instance of being recorded as convicted, cautioned, reprimanded or warned, but excluded Penalty Notices for Disorder, in a one-year period after release from prison and appended to the dataset [35].

### Statistical models

Two explicit assumptions are required to establish a treatment effect for an intervention made in the context of an observational study. The first is to adjust for as many confounders as possible that are associated with treatment assignment and with the outcome. A second is for there to be complete ‘overlap’ in the distribution of prognostics across treatment modalities. When these conditions have been fulfilled then there is ‘strong ignorability’ of how an individual came to be treated relative to the outcome [46]. Rosenbaum and Rubin [46] established a propensity score structure for binary treatment which has been deployed to minimise selection bias [27, 32]. Methodologies encompassing regression models and inverse probability weighting have been developed for the evaluation of multiple treatments [33]. A treatment effect will be assessed using four alternative methods: regression adjustment (RA), inverse probability weighting (IPW), augmented inverse probability weighting (AIPW) and inverse probability weighted regression adjustment (IPWRA).

All four methods yield two sets of estimated quantities: a set of model-based predicted means for each treatment group (known as Potential Outcome Means), which are then compared to find a set pairwise differences between treatment group means (termed Average Treatment Effect). As the outcome measure is binary, predicted treatment means can be interpreted as predicted probabilities of reoffending in each group and their pairwise differences can be interpreted as risk differences.

The RA method first models the reoffending outcome (on a set of prognostics), separately for each treatment, then predicts the probability of reoffending for all subjects in the database, all assigned to each treatment in turn, and averages individual predictions by treatment group. The RA method does not model the treatment assignment. The IPW method first models the treatment assignment (on a set of prognostics), then predicts the probability of treatment assignment for each subject in the database and computes the inverse of these probabilities (termed Inverse Probability Weights, or IPW). The latter are then used when predicting the reoffending outcome for each subject in the database, according to their assigned treatment only, and averages individual predictions by treatment group. The IPW method does not model the reoffending outcome.

The remaining two methods also first model treatment assignment for deriving inverse probability weights as described above, though these weights are used at different stages. The AIPW method models the reoffending outcome, separately for each treatment, then predicts the probability of reoffending for all subjects in the database, all assigned to each treatment in turn, adjusts such predictions using inverse proportional weights as a correction factor, and finally averages individual predictions by treatment group. The IPWRA method differs only in that it uses inverse probability weights when modelling the reoffending outcome, and not as a correction factor of the model-based predictions. So both the AIPW and IPWRA methods are said to be ‘doubly-robust’ because they explicitly model both the treatment assignment and the reoffending outcome; their estimates will be consistent even when either the treatment model or the outcome model (but not both) are mis-specified. Doubly robust methods are considered the most efficient at estimating treatment effects for multiple treatment in observational studies [33].

### Prognostic variables

Information held on prison leavers included socio-demographic details, release dates and spell length. It should be noted that ethnic group is not included in this dataset, rather using a wider ‘nationality’ definition. Preliminary analysis suggested large levels of missing or incomplete data resulting in this variable being dropped from the final analysis. In the UK, criminogenic risk is measured by the Offender Group Reconviction Scale 3 (OGRS), which is an actuarial tool established to predict general reoffending and is administered to all adult prisoners across England and Wales [26]. It incorporates three static risk factors (age, gender and offending history) to derive the likelihood of reoffending 1–2 years from prison discharge and has been shown to achieve a high level of predictive accuracy [26]. OGRS scores are broken down into low, medium, high/very-high risk categories. Use of OGRS scores have been incorporated within a wider Offender Assessment System (OASys) that integrates actuarial risk with dynamic and static risk factors including details of the offence, housing, employment/education/training, financial, relationship, lifestyle/associates, drug and alcohol, emotional well-being, thinking and behaviour, attitudes and general health needs. These components have been used in quasi-experimental studies creating matched control samples [9, 47]. All adult people in prison were assessed for an AUD by the prisoner’s offender manager. If the prisoner’s alcohol consumption was explicitly linked to offending and/or severity of use prior to incarceration was significant, then an AUD was recorded. Severity of alcohol consumption was determined on an ordinal scale with the highest-level equivalent to dependency. Out of 59,150 prison leavers, 26,654 people in prison (45%) were identified as having an AUD.

Characteristics of the baseline sample were determined through a number of static and dynamic prognostic variables derived from the merged dataset. These included socio-demographics (age, gender), offending history, length of prison spell and dynamic variables derived from the OASys actuarial risk assessment including emotional well-being, temper and control, problem solving skills, awareness of criminal consequences, general health status, whether unemployed, access to prosocial activities, illicit drug use, alcohol consumption linked to offending and levels of binge drinking.

### Covariate balance

**Fig. 1:**
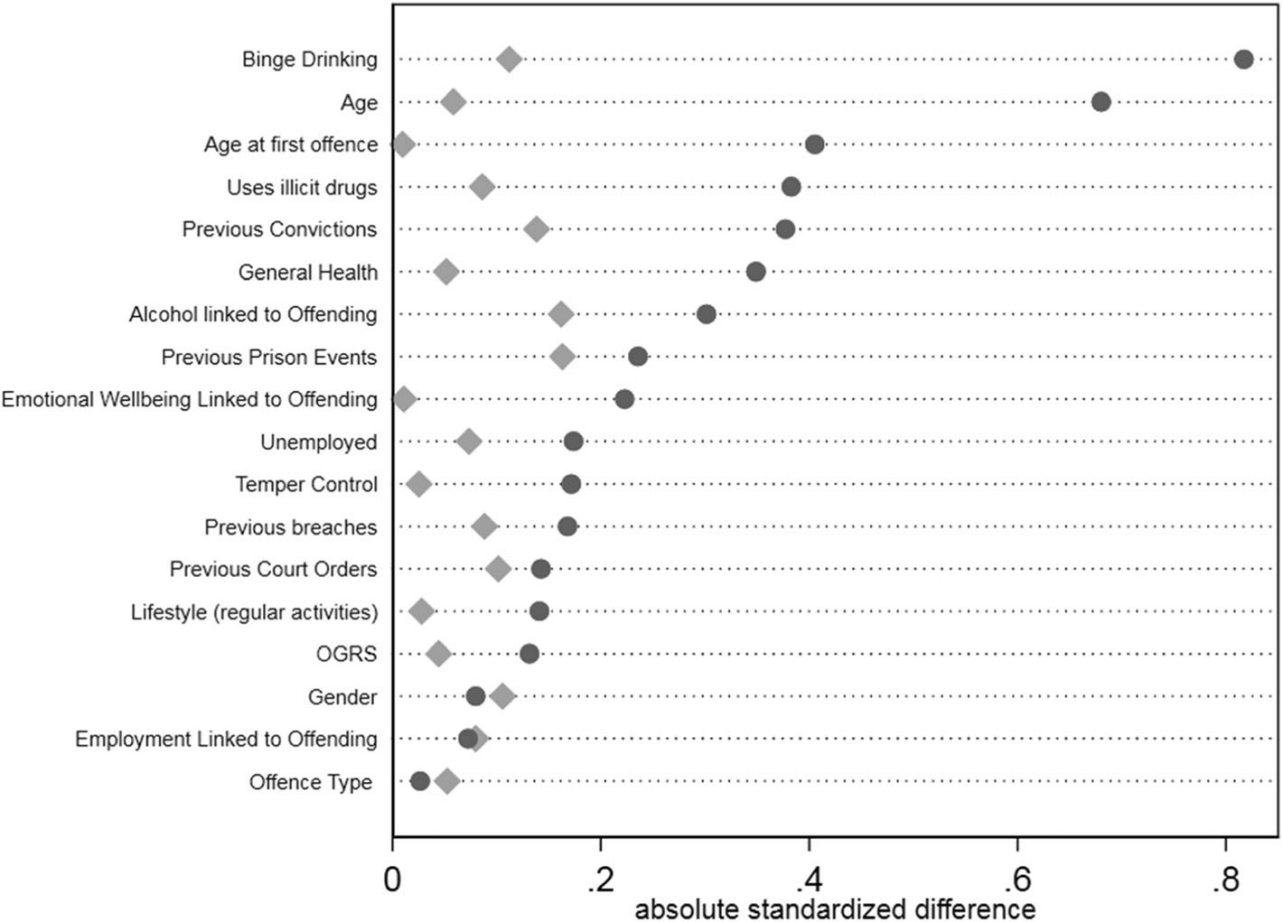
Covariate Balance for Pharmacological Treatment compared to control group. Legend: Dots represent raw or weighted data. Diamonds represent weighted data

**Fig. 2:**
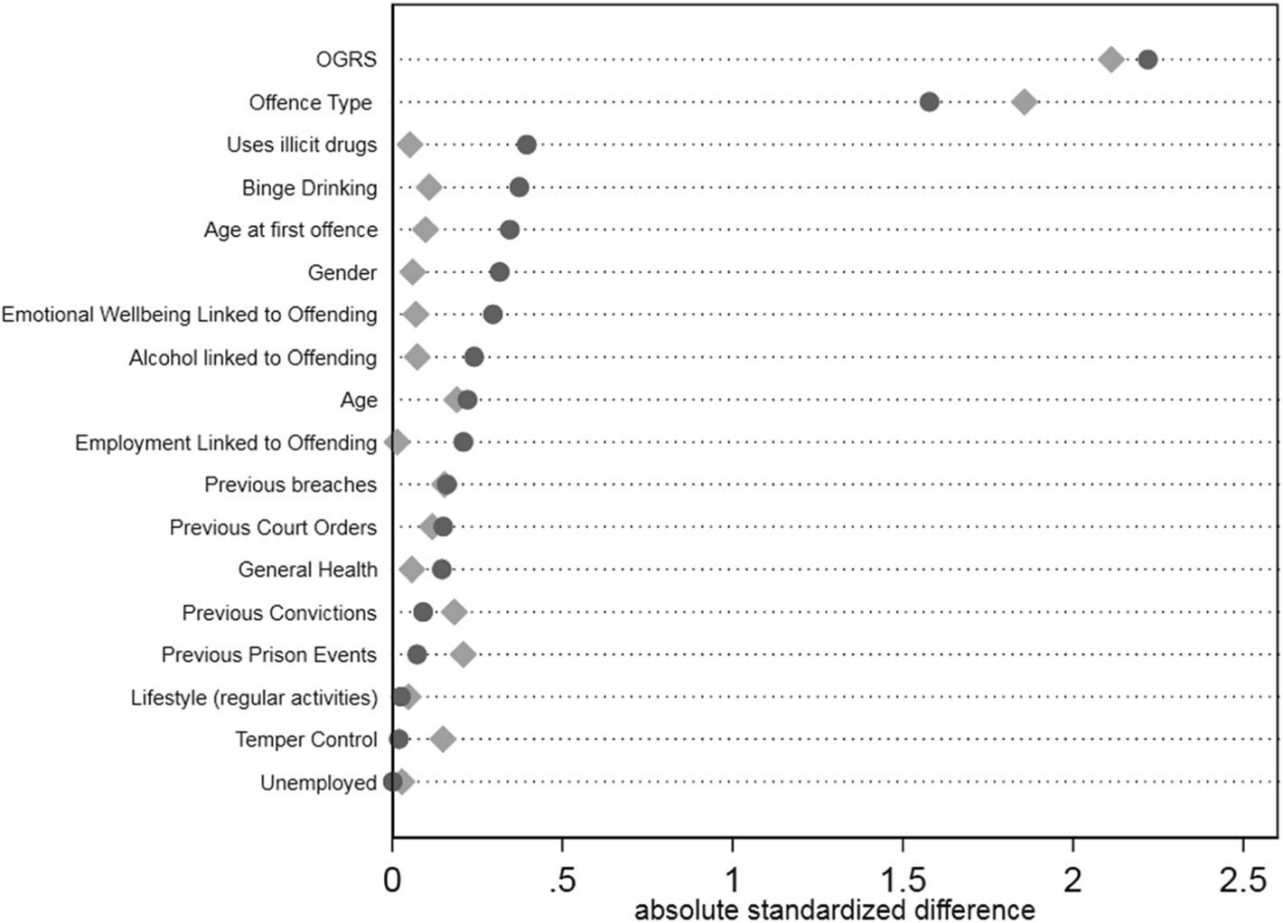
Covariate Balance for RNR compliant Treatment compared to control group. Legend: Dots represent raw or weighted data. Diamonds represent weighted data

**Fig. 3:**
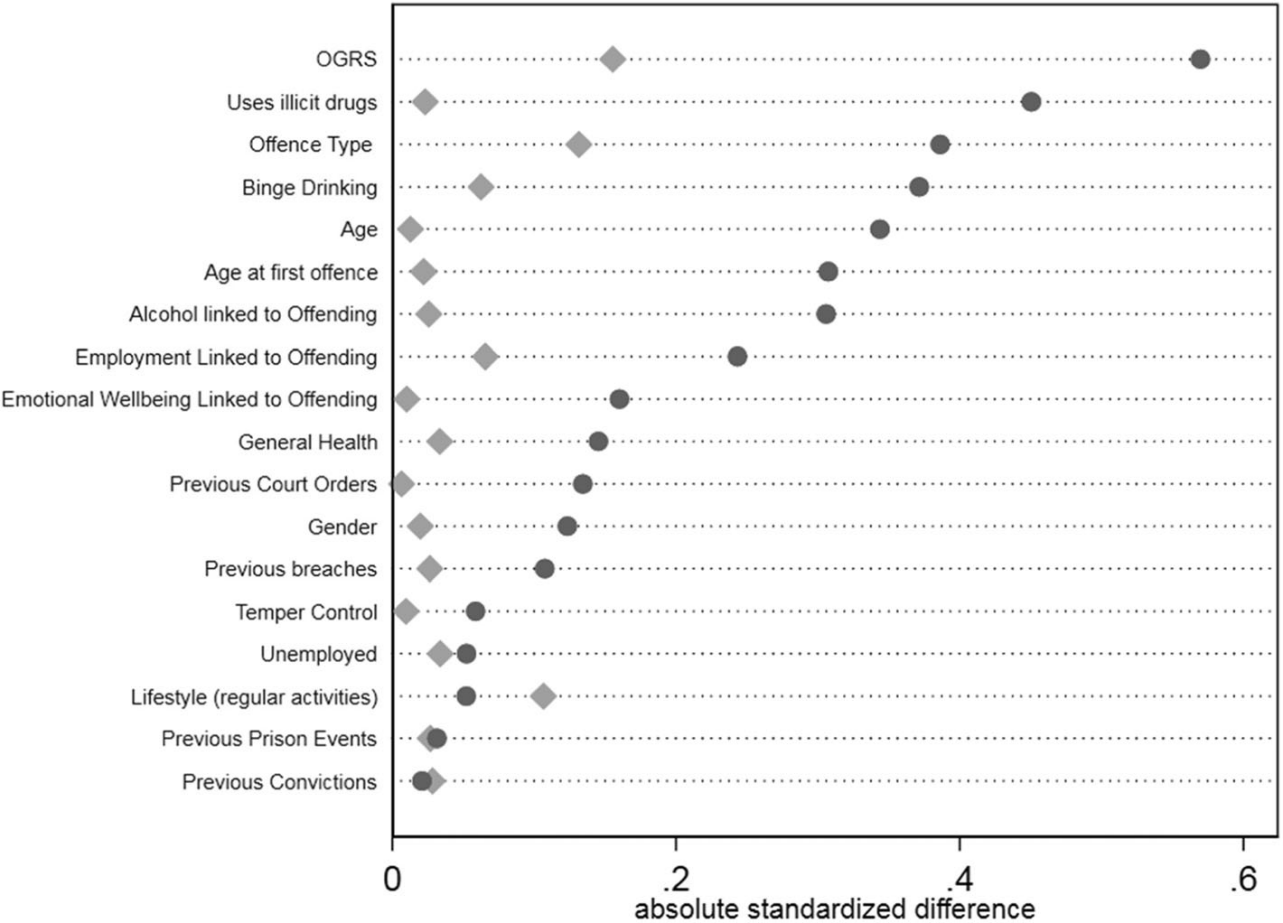
Covariate Balance for PSI Treatment compared to control group. Legend: Dots represent raw or weighted data. Diamonds represent weighted data

Designed studies in which treatments are allocated according to a randomisation schedule are considered to yield unbiased treatment effect estimates, as treated and control groups are considered equivalent across subjects’ characteristics [10]. However, in observational studies treatment selection is often influenced by subject characteristics, resulting in systematic differences in baseline characteristics between subjects in the treated and control groups [48, 49]. The lack of random treatment allocation has been shown to yield biased treatment effect estimates [5]. A key requirement of analytical techniques for estimating treatment effect in observational studies is therefore to try re-balance the subjects’ characteristics between groups being compared, termed “covariate balance”. As regression adjustment does not use weights to derive an estimate and the other estimates use the same weights, only one covariate summary is required for balancing. The extent to which the weighting method achieves covariate balance was assessed visually using the approach recommended by Austin and Stuart [4]. Their suggestion is to illustrate absolute mean standardised differences between covariates in each treatment group relative to the control group, both before (“raw”) and after (“weighted”) the weighting process. Ideally, all symbols of the weighted absolute mean standardised differences would be aligned above the value of zero on the horizontal axis. For pharmacological treatment (Fig. 1), three variables (gender, employment related to offending and offence) were unbalanced after weighting. For the RNR compliant group (Fig. 2), five covariates (offence, employment linked to offending, previous convictions, previous prison events and temper control) were less balanced after weighting than before it, very likely due to the small samples size of this group. Figure 3 shows two prognostics (lifestyle and previous convictions) less balanced after weighting than before it for the PSI group. Sensitivity tests were run removing the unbalanced variables which showed no difference in outcomes.

## Results

Table 2 presents the crude reconviction rates for the treated and control populations using the derived treatment modalities. RNR compliant treatment recorded the lowest rate of reoffending (36.5%) of all treatment modalities, i.e., pharmacological treatment (53.8%) and PSIs (40.2%), and also showing reoffending rates lower than the untreated group (43.7%).

**Table 2:** Unweighted reoffending rates of people in prison receiving SAT treatment and control group

Treatment group	Did not reoffend		Reoffended		Total
	%	N	%	N	
Control	56.3	13,510	43.7	10,497	24,007
Pharmacological Treatment	46.2	339	53.8	394	733
RNR compliant	63.5	153	36.5	88	241
PSI	59.8	1000	40.2	673	1673

Table 3 examines the treatment effect for each treatment modality using the four models. As expected, the larger the sample size, the less variability in the estimated reoffending rate, which was noticeable for the control group. Different methods yielded similar estimates for each treatment modality, except for the IPWRA method, which for RNR compliant treatment yielded an estimate of 0.756 compared to a range of 0.342 to 0.417 from the other three methods. A diagnostic look at the coefficients from the logistic regression models in the RNR compliant treatment revealed a malfunction called ‘convergence failure’ [3] in both the model for the reoffending outcome and for the treatment allocation. Therefore, the predicted reoffending rate of 0.756 for the RNR compliant group from the IPWRA method was deemed an unreliable estimate, with it being the highest of the four groups being compared and more than twice its crude rate of 0.365. Instead, across the other three methods (AIPW, IPW and RA) exposure to RNR compliant treatment consistently yielded the lowest estimated re-offending rate of all four groups compared.

**Table 3:** Weighted Treatment Effects (Potential Outcome Means) of people in prison receiving multiple component treatment compared to a control group by treatment effect estimator

	Estimated Reoffending Rate			
Treatment group	IPWRA	AIPW	IPW	RA
Control	0.435	0.435	0.436	0.436
Pharmacological Treatment	0.514	0.527	0.531	0.505
RNR compliant	0.756	0.417	0.342	0.412
PSI	0.450	0.450	0.424	0.441

Table 4 presents the risk differences between predicted reoffending rates for all treatment modalities, by each estimation method. We did not interpret the results for the RNR compliant group from the IPWRA method, given the malfunction of its underlying logistic regression model. 99% coverage was needed to yield a set of 95% simultaneous confidence intervals [11]. A single-step Bonferroni adjustment for multiplicity was applied to *p*-values from z-tests [20]. The RA method, which unlike the other three methods does not use a weighting scheme, yielded no statistically significant result among the six risk differences. Results from the other three methods suggested an overall higher reoffending rate of the pharmacological treatment only group compared to the control group, with a statistically significant effect from both AIPW method (*p*-value = 0.0279, risk difference = 0.092, 95% CI [0.008, 0.0176]) and IPWRA method (*p*-value = 0.0369, risk difference = 0.079, 95% CI [0.005, 0.154]). Only the IPW method yielded a statistically significant reduction in reoffending rate for the RNR compliant group compared to the pharmacological treatment only group (*p*-value = 0.0072, risk difference = 0.188, 95% CI [0.039, 0.339]). Despite the reoffending rate for the RNR compliant group being the lowest estimate for all four groups also from the RA and AIPW methods, the latter did not yield significant risk differences between the RNR compliant group and any other group. This result can be explained by the small sample size of the RNR compliant group being the limiting factor for the precision of its estimates.

**Table 4:** Pairwise risk differences (Average Treatment Effect), Bonferroni adjusted across multiple treatments and comparison group, by estimating method

Estimating method	Risk Difference	Standard Error	Z statistic	Adjusted *p*-value	99% Confidence Interval	
**RA**						
Control vs Pharmacological Treatment	−0.07	0.03	−2.33	0.118	− 0.147	0.007
Control vs RNR	0.023	0.154	0.15	1	−.374	0.42
Control vs PSI	− 0.005	0.017	−0.33	1	− 0.05	0.04
Pharmacological Treatment vs RNR	0.093	0.157	0.59	1	−.0311	0.498
Pharmacological Treatment vs PSI	0.064	0.034	1.86	0.377	−0.024	0.153
RNR vs PSI	−0.029	0.155	0.19	1	−0.43	0.371
**IPW**						
Control vs Pharmacological Treatment	−0.095	0.037	−2.57	0.061	−0.190	0.0002
Control vs RNR	0.094	0.045	2.07	0.231	−0.023	0.211
Control vs PSI	0.012	0.018	0.68	1	−0.034	0.059
Treatment vs RNR	0.188	0.058	3.24	0.0072*	0.039	0.339
Pharmacological Treatment vs PSI	0.107	0.041	2.62	0.0528	0.002	0.213
RNR vs PSI	−0.081	0.048	−1.68	0.558	−0.207	0.044
**AIPW**						
Control vs Pharmacological Treatment	−0.092	0.032	−2.83	0.0279*	−0.176	−0.008
Control vs RNR	0.017	0.153	0.12	1	−0.377	0.412
Control vs PSI	−0.015	0.018	−0.85	1	−0.062	0.031
Pharmacological Treatment vs RNR	0.109	0.156	0.7	1	−0.294	0.513
Pharmacological Treatment vs PSI	0.076	0.037	2.07	0.2307	−0.018	0.171
RNR vs PSI	−0.033	0.154	0.21	1	−0.43	0.364
**IPWRA**						
Control vs Pharmacological Treatment	−0.079	0.029	−2.74	0.0369*	−0.154	−0.005
Control vs RNR	−0.321	0.100	−3.19	0.0085*	−0.580	−0.062
Control vs PSI	−0.015	0.018	−0.85	1	−0.061	0.031
Pharmacological Treatment vs RNR	−0.241	0.104	−2.31	0.125	−0.510	0.028
Pharmacological Treatment vs PSI	0.064	0.034	1.89	0.352	−0.023	0.151
RNR vs PSI	0.305	0.102	2.99	0.0167*	0.042	0.568

*p < 0.05;

## Discussion

This study assesses a treatment effect on recidivism as measured by post-treatment reconviction rates for multiple component correctional SAT. SAT was divided into three groups relating to pharmacological treatment only (28%), RNR compliant treatment (9%) and PSIs (63%). The weighted reoffending rates for people in prison treated for an AUD-only was estimated to be between 34 and 42% which is lower than the 48% overall offending rates for adults released from custody [36].

Using methods for estimating a multivalued treatment effect from an observational study, it was possible to establish that RNR compliant approaches reported lower offending rates than the control group. Other treatments including PSI and pharmacological only approaches did not reduce offending rates relative to the control group. This study adds to the wider literature on a treatment effect on recidivism for substance misusers. Systematic reviews [13, 15] suggest that for any substance misused (illicit drugs and alcohol) some treatment types can realise significant reductions in recidivism. However, direct comparisons to other studies are difficult due to the heterogeneity of populations measured methods used, varying definitions of recidivism and sample attrition [15].

The study also finds that pharmacological only treatment for an AUD leads to a significant increase in reoffending relative to the control group. This is contrary to the findings from a systematic review [13] that found the majority of pharmacological interventions for substance misusers were likely to report reductions in reoffending. This may be in part explained by differing methodological approaches, but this study suggests that use of pharmacological only treatment is likely to be an unintended consequence of operationalising SAT within an English correctional setting. Guidance suggests that PSIs should be delivered alongside pharmacological treatment [14]. People in prison receiving a short sentence or housed for a short spell, the availability of PSIs and an emphasis on treating the health-related consequences of alcohol consumption may have resulted in PSIs not being delivered despite services’ best intentions. It is also likely that receipt of pharmacological treatment-only reflected the severity of alcohol-related needs that required a clinical response prior to any cognitive behavioural work. This has implications for the findings as we suggest that addressing this unintended consequence, by sequencing PSIs into SAT may have a positive effect on reconviction rates.

Therefore, we suggest that the principle of treatment ‘equivalence of care’ has reframed English correctional SAT towards delivering health-based approaches commensurate to community-based treatment. Structured programs and interventions designed on RNR programming principles have been subsumed within more health-focused approaches. Guidance documents have emphasized medical management with the assumption that “drug treatment significantly reduces drug-related crime” ([14]; 13). The universal principle of ‘equivalence of care’ has made operationalising a national system treatment system that incorporates risk of offending within a medicalised model of treatment to be highly problematic. Studies in the UK on smaller scale offender behavioural programs found difficulties in ensuring risk was adequately calibrated to need and dosage [24]. As PSIs become focused on managing relapse or minimising the harm from excessive alcohol consumption, there is a likely dilution of an effect on reoffending [16, 43]. PSIs form part of the therapeutic delivery model but these interventions are often not delivered due to the difficulties of operationalising interventions in a correctional setting [28]. Contextually, [29] [30]) extend this argument to suggest that the prisons operate with a command-and-control, hierarchical management structure and an overarching suspicion of public health approaches will also further limit the ambition of delivering genuine equivalence of care in prison healthcare.

The deployment of RNR principles has also been shown to be problematic where multiple treatments coexist. Operationalising severity of substance misuse to encompass dependence and how consumption changes over time has been shown to be challenging [50]. The low number of RNR compliant interventions may be a reflection of the difficulty of operationalising this approach in correctional settings. Marlowe [34] argues that treating both non-dependent and low-risk substance misusers may also dilute a treatment effect on recidivism. The delivery of treatment within correctional settings has also been viewed as problematic as few people in prison may access the treatment they need, using non-evidence based interventions such as group-based drug education [41] and what interventions are available may be delivered poorly [34]. Evidence from English criminal justice settings suggest that the majority of PSIs are limited in scope with little therapeutic contact, focusing on administrative functions [6, 7].

There is also a suggestion that some substance misuse practitioners are resistant to treating high-risk people in prison as they are more likely to be disruptive and lack motivation to change [51]. Critics also argue that prison-based treatment will include people in prison with tangential needs (i.e. involvement in activities relating to the production or supply of substances or with high rates of co-occurring disorders including suicidal thoughts, anxiety and depression, and generally in poor physical health). Clinical screening tools may also overstate the need for treatment by focusing on a prisoner’s immediate severity of use, and the role of self-selection through self-referrals or court-mandated treatment creating selection biases [31]. Despite these challenges, correctional treatment for AUDs provides an opportunity for breaking the cycle of substance misuse and offending if effectively planned and integrated across health and criminal justice jurisdictions. Integrating approaches across offender managers and public health can also enhance the detection and diagnosis of alcohol-related needs. This can create improved treatment pathways that can reduce the likelihood of people in prison with an AUD receiving pharmacological only treatment. More depth is also required to understand the relationship between reoffending and relapse [43]. A focus on a single measurement (reoffending) is unlikely to give a full picture of a treatment effect. More quantitative work is required to situate changes in offending patterns post release with other clinical or recovery-orientated measures [53].

The evidence from this study suggests there is an opportunity to address the issues underpinning the delivery models to develop an integrated public health and criminal justice-orientated approach that merges pharmacological treatment with RNR principles into a coherent, unified treatment system. We suggest that correctional treatment for AUDs should not be seen in binary public health or criminal justice terms, rather there is a need to enhance treatment models to deliver evidence-based practice for to support both clinical and crime reduction needs. We further suggest extending this approach to assessing a treatment effect for illicit drug misusers in prison, and for people in prison who misuse illicit drugs and alcohol in combination.

### Strengths and limitations

This study examines the effectiveness of multiple treatment systems in reducing recidivism for people in prison who have been diagnosed with AUD. The value of this study is that it simultaneously compares treatments to each other and to a control group using methods developed for estimating a multicomponent treatment effect from an observational study. The multiple treatment and control groups have been shown to be better balanced after reweighting using these methods (Figs. 1, 2 and 3). The study also has a national geographic focus (England) and includes large sample sizes. However, some limitations should be noted. Firstly, there is limited information available on the detail within each treatment component, what the intervention comprised of, when it was delivered and how relevant it was to their needs. It is likely that broad categorisations do not capture the nuance of the treatment experience in each prison [12]. Moreover, the categorisation of RNR compliant treatment was likely a coding construct as the treatment system did not specifically code for this intervention. Although this will include some specifically designed RNR programs [23] it is also likely that some of the people in prison accessing treatment will be unaware that are receiving RNR treatment. Second, while alcohol severity was determined, the scales used were not validated tools. Covariate balance was achieved by measuring how strongly alcohol use was associated with reoffending. More sophistication of measuring differential levels of severity is required to ensure the treatment groups are balanced effectively.

The study has explored the use of regression-based methods for estimating treatment effect on recidivism rates in an observational study context. It is of note that one of the “doubly-robust” methods did not work as anticipated, contrary to what was expected in recent methodological literature [33]. This study has suggested that caution is advised in presence of groups with relatively small sample sizes. The small sample size of the RNR group caused its multinomial logistic model of the IPWRA method to malfunction, yielding an estimated recidivism rate much higher than its observed crude rate. Such malfunction may be avoided by gathering a larger sample size in the problem group, but this being an observational study, group sample sizes were fixed. The problem was examined by comparing results from across the four statistical analysis methods deployed. It is therefore recommended that similar studies should report results from a range of statistical methods. This will minimise the risk of relying on estimates from a single modelling technique, which may be revealed as unreliable only when compared to estimates of the same quantity from other similar techniques. Consequently, we recommend that future work should routinely report on a range of estimated rates resulting from using more than one statistical methodology. The outcome measure for this study is binary (whether a person offended or not) and lacks a nuanced understanding of differential levels of reoffending by offence type. It is possible for example, that changes were noted by type of crime committed. Further work is required to develop multimodal outcome measurements that include change in the nature and severity of offence committed. Finally, although offender programming was decommissioned, it is possible that the control group may have received some legacy interventions for their wider offending needs, although it is anticipated that this number would be small. Consequently, these subjects may have had a non-substance misuse specific treatment effect on reducing recidivism.

## Conclusion

The modelling of the effect of each treatment component suggests that RNR compliant treatment could be the most effective intervention compared to other treatments. RNR compliant treatment was the only intervention arm that, except for one method (IPWRA), consistently yielded the lowest estimated rate of reoffending of all groups being compared. However, in contrast, pharmacological only interventions demonstrated statistically significant negative results (i.e. increased rate of reoffending) compared to the control group which may be attributed to the unintended consequence of establishing equivalence of care in custodial settings with community services. It is suggested that prioritisation of equivalence of care has emphasized pharmacological interventions sometimes at the expense of an integrated approach that incorporates use of PSIs. There is an opportunity to develop an integrated approach to meeting the needs of people in prison with an AUD in ensuring an equitable public health and criminal justice response.

## Data Availability

The datasets generated and analysed during the current study are not publicly available as they are owned by the Ministry of Justice and Public Health England and is considered restricted information.

## Abbreviations

AIPW: Augmented inverse probability of treatment weighting
ATE: Average treatment effect
AUD: Alcohol use disorder
CBT: Cognitive behavioural therapy
IPW: Inverse probability of treatment weighting
IPWRA: Inverse probability of treatment weighted regression adjustment
NDTMS: National Drug Treatment Monitoring System
NHS: National Health Service
OASys: Offender Assessment System
OGRS: Offender Group Reconviction Scale
PSI: Psychosocial interventions
RA: Regression adjustment
RNR: Risk Need Responsivity
SAT: Substance abuse treatment
